# Azithromycin treatment response as a probe to attribute bacterial etiologies of diarrhea using molecular diagnostics: a reanalysis of the Antibiotics for Children with Severe Diarrhea (ABCD) trial

**DOI:** 10.3389/fmicb.2025.1606207

**Published:** 2025-05-20

**Authors:** Jennifer Cornick, Sarah Elwood, James Platts-Mills, Patricia Pavlinac, Karim Premji Manji, Christopher R. Sudfeld, Christopher P. Duggan, Queen Dube, Naor Bar-Zeev, Karen Kotloff, Samba O. Sow, Sunil Sazawal, Benson Singa, Judd L. Walson, Farah Naz Qamar, Tahmeed Ahmed, Ayesha De Costa, Elizabeth T. Rogawski McQuade

**Affiliations:** ^1^Malawi Liverpool Wellcome Trust Research Programme, Queen Elizabeth Central Hospital, Blantyre, Malawi; ^2^Department of Clinical Infection, Microbiology and Immunology, Institute of Infection, Veterinary and Ecological Sciences, University of Liverpool, Liverpool, United Kingdom; ^3^Division of Infectious Diseases and International Health, Department of Medicine, University of Virginia, Charlottesville, VA, United States; ^4^Department of Global Health, University of Washington, Seattle, WA, United States; ^5^Department of Epidemiology, University of Washington, Seattle, WA, United States; ^6^Department of Pediatrics and Child Health, Muhimbili University of Health and Allied Sciences, Dar es Salaam, Tanzania; ^7^Department of Global Health and Population, Harvard T.H. Chan School of Public Health, Boston, MA, United States; ^8^Division of Gastroenterology, Hepatology and Nutrition, Boston Children’s Hospital, Boston, MA, United States; ^9^International Vaccine Access Center, Johns Hopkins Bloomberg School of Public Health, Baltimore, MD, United States; ^10^Center for Vaccine Development and Global Health, Department of Pediatrics, University of Maryland School of Medicine, Baltimore, MD, United States; ^11^Center for Vaccine Development and Global Health, Department of Medicine, University of Maryland School of Medicine, Baltimore, MD, United States; ^12^Centre pour le Développement des Vaccins, Bamako, Mali; ^13^Center for Public Health Kinetics, New Delhi, India; ^14^Childhood Acute Illness and Nutrition Network, Nairobi, Kenya; ^15^Kenya Medical Research Institute, Nairobi, Kenya; ^16^Department of International Health, Medicine and Pediatrics, Johns Hopkins University, Baltimore, MD, United States; ^17^Department of Pediatrics and Child Health, Aga Khan University, Karachi, Pakistan; ^18^Nutrition and Clinical Services Division, International Centre for Diarrhoeal Disease Research, Dhaka, Bangladesh; ^19^Department of Maternal, Child, and Adolescent Health and Aging, World Health Organization, Geneva, Switzerland; ^20^Department of Epidemiology, Rollins School of Public Health, Emory University, Atlanta, GA, United States

**Keywords:** diarrhea, azihtromycin, etiology, children, *Shigella*, RT-PCR

## Abstract

**Background:**

Multi-pathogen molecular diagnostics enhance our understanding of the pathogen-specific burden of diarrhea. However, attributing etiology remains challenging in high-burden settings where coinfections are common. The Antibiotics for Children with severe Diarrhea (ABCD) trial provides a unique opportunity to leverage azithromycin treatment response to identify bacterial diarrhea.

**Methods:**

We analyzed data from 6,692 children with watery diarrhea enrolled in ABCD (2017 to 2019) who were randomized to receive azithromycin or placebo. We modelled the heterogeneity in the azithromycin treatment response by the enteric pathogen quantity detected by quantitative PCR using log-binomial regression.

**Results:**

Azithromycin treatment response varied by pathogen quantity, with the strongest effect observed for *Shigella*. Each log₁₀ increase in *Shigella* quantity was associated with a 13% reduction (95% CI: 3–23%) in diarrhea risk at day 3 in the azithromycin group compared to placebo. We observed similar, though non-significant, trends for *Vibrio cholerae*, ST-ETEC, and tEPEC. In contrast, no association was found between pathogen quantity and azithromycin response for *Campylobacter*, LT-ETEC, or EAEC. These patterns remained consistent when evaluating hospitalization or death risk within 90 days.

**Conclusion:**

The observed associations between azithromycin treatment response and pathogen quantity for *Shigella*, *Vibrio cholerae*, ST-ETEC, and tEPEC support prior evidence that these pathogens are likely causes of diarrhea when present in high quantities. Conversely, the absence of a similar response pattern for *Campylobacter*, LT-ETEC, and EAEC is consistent with large-scale studies showing a limited association between their quantities and diarrhea.

## Introduction

Diarrheal diseases are the fifth leading cause of death in children under five, with the highest burden of disease experienced in low- and middle-income countries (LMICs) (Troeger et al., 2018). Accurate estimates of the burden of specific etiologies of diarrhea are helpful for global resource allocation and policy making and may be useful to inform appropriate treatment measures beyond the WHO IMCI recommended management (WHO, 2014). Quantitative PCR (qPCR) diagnostics have been used to attribute likely diarrhea etiologies by leveraging comparisons of pathogen quantity between diarrhea cases and non-diarrheal controls in multiple large global studies, including the Global Enteric Multicenter Study (GEMS) (Liu et al., 2016), the Malnutrition and the Consequences for Child Health and Development (MAL-ED) study (Platts-Mills et al., 2018), and the Global Pediatric Diarrhea Surveillance network (Cohen et al., 2022).

The Antibiotics for Children with severe Diarrhea (ABCD) study, a 7-country, randomized, double-blinded, placebo-controlled trial, assessed if a 3-day course of azithromycin reduced mortality or improved linear growth among children with acute watery diarrhea accompanied by dehydration or undernutrition (Ahmed et al., 2021). In the study, rotavirus (21.1%) was the leading cause of diarrhea, while 28.3% of diarrheal cases had a likely bacterial etiology, most commonly *Shigella*, enterotoxigenic *E. coli* encoding heat stable enterotoxin (ST-ETEC), and typical enteropathogenic *E. coli* (tEPEC) (Pavlinac et al., 2024). While the impact of azithromycin among all enrolled children on mortality was minimal, application of molecular diagnostics suggested that azithromycin was effective at reducing risk of day 3 diarrhea and day 90 hospitalization or death among children with likely bacterial diarrhea (Pavlinac et al., 2024). Due to the absence of diarrhea-free controls in ABCD, pathogen-specific quantity cut-offs were applied to assign etiology, based on the strong associations of those quantities with diarrhea previously observed in MAL-ED and GEMS (Liu et al., 2016; Platts-Mills et al., 2018). However, there was a residual benefit of azithromycin among episodes with bacteria detected at a lower quantity. For example, children in ABCD with a likely bacterial etiology had a 3.1% absolute reduction in risk of 90-day hospitalization or death when treated with azithromycin compared to a 2.3% reduction among children with bacteria detected but not attributed (Pavlinac et al., 2024). These findings suggest the previously used cut-offs likely missed true bacterial episodes that also responded to treatment.

The ABCD study offers a unique opportunity to further investigate diarrheal etiology using azithromycin treatment response as a probe. Azithromycin is a broad-spectrum antibiotic with known efficacy against Gram-negative pathogens, including pathogenic *E. coli*, (Sanders et al., 2007) *Campylobacter* spp., (Vukelic et al., 2010) and *Shigella* spp. (Khan et al., 1997; Basualdo and Arbo, 2003). We therefore expect an azithromycin treatment response in diarrhea cases in which bacteria are the true etiological agent.

Here we investigate if heterogeneity in the treatment response by pathogen quantity can inform the assignment of diarrhea etiology and potentially refine etiological quantity cut-offs for enteric bacteria if a quantitative threshold for the treatment response is observed.

## Methods

The ABCD study design (Alam et al., 2020), primary analysis results (Ahmed et al., 2021), and post-hoc analyses incorporating qPCR enteric pathogen testing (Pavlinac et al., 2024) have been previously described. Briefly, 8,268 children 2–23 months of age were enrolled between June 2017 and July 2019 from seven sites in Bangladesh, India, Kenya, Malawi, Mali, Pakistan, and Tanzania if they presented to study hospitals with acute watery diarrhea. Eligible children had some or severe dehydration and/or moderate wasting or severe stunting, as previously defined (Alam et al., 2020). Children who had received antibiotics in the 2 weeks prior to presentation or with clear indications for antibiotic treatment (i.e., dysentery, severe acute malnutrition, signs of other infections requiring antibiotics) were excluded.

Participants were randomized to receive either 3 days of azithromycin (10 mg/kg/day) or placebo. Before randomization, study staff collected either whole stool or a flocked rectal swab. Enteric pathogens were tested via qPCR in the first approximately 1,000 children enrolled at each site using the TaqMan array card platform as described previously (Alam et al., 2020). Cycle thresholds (Ct’s) derived from rectal swabs were adjusted by the pathogen-specific mean cycle threshold difference between paired rectal swabs and whole stools to account for differences in sample type. Pathogens detected were adenovirus 40/41, astrovirus, *Campylobacter jejuni*/*coli*, *Cryptosporidium*, *Enterocytozoon bieneusi*, *Giardia*, norovirus GII, rotavirus, sapovirus, *Shigella*/enteroinvasive *Escherichia coli* (EIEC), *Vibrio cholerae*, heat-stable enterotoxigenic *E. coli* (ST-ETEC), heat-labile enterotoxigenic *E. coli* (LT-ETEC), typical enteropathogenic *E. coli* (tEPEC), and enteroaggregative *E. coli* (EAEC). Follow-up visits were conducted to ascertain presence of diarrhea on day 3 after enrolment and rehospitalization and vital status 90 days post-enrollment.

Pathogens were analyzed using the continuous Ct as a marker of relative pathogen quantity. Co-etiologies (i.e., coinfections at a quantity associated with diarrhea) were defined based on pathogen-specific qPCR Ct cut-offs derived for ABCD (Pavlinac et al., 2024) from two large multisite studies of diarrhea that included non-diarrheal controls, GEMS (Liu et al., 2016) and MAL-ED (Platts-Mills et al., 2018). A co-etiology was defined as the detection of any other pathogen at a quantity above (or equally, a qPCR Ct below) the etiological cut-off. The primary outcome assessed in this analysis was presence of diarrhea 3 days after enrolment, selected to maximize power to detect treatment effect heterogeneity and to be consistent with prior analyses (Pavlinac et al., 2024). The combined outcome of rehospitalization or death by 90 days after enrolment was the secondary outcome.

We modelled the heterogeneity in the azithromycin treatment effect by pathogen quantity detected using log-binomial regression for pathogens previously associated with diarrhea (Liu et al., 2016) or with at least 5% prevalence. The model adjusted for the day of diarrhea on which the sample was collected and co-detection of other pathogens. Co-detection was specified with two variables representing the sum of the episode-specific attributable fractions (AFes) for all bacteria and the sum of the episode-specific AFes for all viruses and protozoa. AFes were estimated by plugging in the observed pathogen quantities in ABCD to site-specific attribution models previously developed in GEMS and MAL-ED and taking the median of 1,000 estimates drawn equally from each of the site-specific models (Cohen et al., 2022). We first included an interaction term between treatment arm assignment and the quantity of pathogen detected when specified with a linear and quadratic term. We conducted a linear trend test for each interaction using the Wald-based *p*-value for the quadratic interaction term with alpha = 0.05. Because the test was not statistically significant for 13 of 15 pathogens evaluated (Supplementary Table S1), we proceeded with modelling the interactions with only linear terms for pathogen quantity across all pathogens for interpretability and comparability. For the diarrhea on day 3 outcome, we further stratified the models by presence of a bacterial co-etiology.

We report the model-predicted pathogen quantity and treatment arm-specific risk of each outcome conditional on sample collection at day 0 and no co-detections using ggpredict from the ggeffects package v. 1.2.1 in R v. 4.2.1. We report the risk ratio for azithromycin compared to placebo for each outcome by pathogen quantity using the interplot 0.2.3 package, both overall and stratified by presence of a bacterial co-etiology for the diarrhea on day 3 outcome. These data were summarized by reporting the change in the effect of azithromycin per each log10 increase in pathogen quantity (i.e., the ratio of risk ratios) based on the interaction term between pathogen quantity and azithromycin. To estimate the proportion of episodes that could be attributed to each bacterial pathogen based on the azithromycin treatment response, we calculated population attributable fractions from these episode-specific risk ratios for day 3 diarrhea. Specifically, to remove azithromycin effects unrelated to the pathogen detected, we divided each episode-specific risk ratio by the largest risk ratio when pathogen quantity was zero (RR_adj_). The population attributable fraction was then calculated as $\sum_{i=1}^{n}1-{\mathrm{RR}}_{{\mathrm{adj}}_{i}}$ (since azithromycin is protective) where *i* included all episodes in which the pathogen was detected at any quantity. Adjusted risk ratios were constrained to be less than or equal to 1 such that the episode attributable fraction could not be negative.

Finally, we calculated the proportion of diarrhea episodes across the range of cycle threshold values that would be treated with antibiotics if a given pathogen-specific cycle threshold were chosen as the cut-off to define etiology and assign treatment (i.e., the proportion of episodes with a cycle threshold value at the cut-off or lower). This proportion was calculated among diarrhea episodes overall to quantify the proportion of all diarrhea episodes that would be treated using a given cut-off and among diarrhea episodes with the pathogen detected at any quantity to quantify the proportion of all potentially pathogen-attributable episodes that would be treated.

## Results

We included 6,692 children from the seven ABCD trial sites who had a valid qPCR result, representing 80.9% of all enrolled participants (*n* = 8,268). The distribution of pathogen quantities detected was similar between the azithromycin and placebo groups (Supplementary Figure S1).

**Figure 1:**
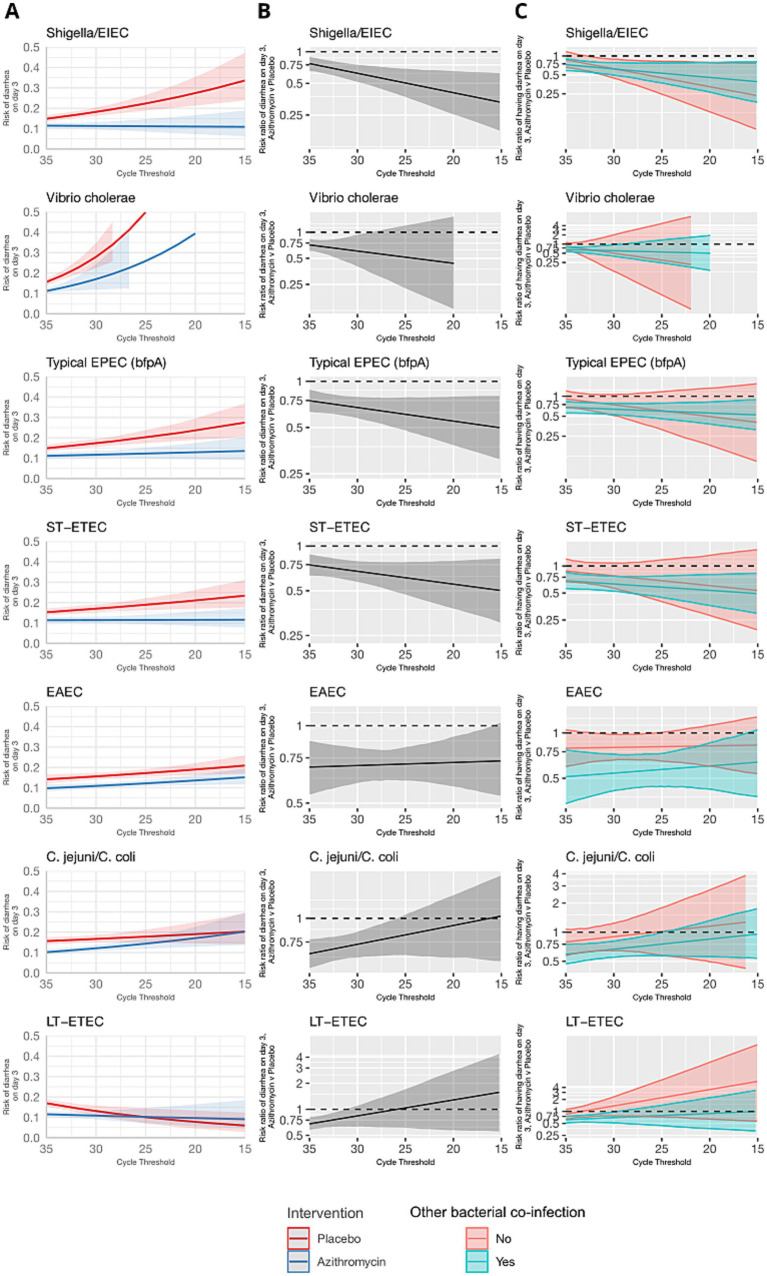
Modification of the effect of azithromycin on the risk of diarrhea on day 3 by bacterial pathogen quantities detected. For each bacterial pathogen, **(A)** the model-predicted risk of diarrhea on day 3 by pathogen quantity and treatment arm (red = placebo; blue = azithromycin), conditional on sample collection at day 0 and no co-detections. **(B)** The risk ratio for azithromycin compared to placebo for diarrhea on day 3 by pathogen quantity adjusted for the day of diarrhea on which the sample was collected and co-detection of other pathogens. **(C)** The adjusted risk ratio for azithromycin compared to placebo for diarrhea on day 3, stratified by the presence of a bacterial co-etiology (green = yes; red = no). Pathogen quantity is specified by the cycle threshold value from qPCR (i.e., smaller cycle thresholds correspond to higher pathogen quantity detected). Bands on all plots indicate 95% confidence bands.

The overall risk of diarrhea on day 3 was 10.2% and was higher in the placebo group (12.0%) compared to azithromycin group (8.4%) (risk ratio: 0.70, 95% CI: 0.61, 0.82). Azithromycin provided stronger protection against diarrhea as pathogen quantity detected increased for a subset of enteric bacteria, specifically *Shigella*, ST-ETEC, tEPEC, and *Vibrio cholerae* on day 3. In the placebo group, diarrhea risk on day 3 increased with pathogen quantity, whereas in the azithromycin group, risk was less dependent on pathogen quantity (Figure 1A). Correspondingly, as pathogen quantities increased the effect of azithromycin became stronger (i.e., risk ratios further from the null) (Figure 1B). The heterogeneity in azithromycin effect by pathogen quantity was most pronounced and statistically significant only for *Shigella*. The risk ratio for diarrhea on day 3 for azithromycin compared to placebo was more protective, specifically 13% (95% CI: 3, 23) lower (i.e., further from the null), per log10 increase in *Shigella* quantity detected (ratio of risk ratios; Table 1). This translated to detection at a high quantity (Ct = 25) associated with a 50% reduction (95% CI: 30, 64) in diarrhea on day 3 compared to a 40% reduction (95% CI: 33, 75) associated with detection at Ct = 30. A similar pattern was observed for *V. cholerae*, ST-ETEC, and tEPEC, though the effect heterogeneity was not statistically significant for these pathogens (Table 1). When stratifying by whether there was a bacterial co-etiology, the association between pathogen quantity and azithromycin treatment response was slightly stronger for episodes in which there was no co-etiology (Figure 1C).

**Table 1:** Change in the effect of azithromycin on risk of diarrhea on day 3 and risk rehospitalization or death by day 90 for each log10 increase in pathogen quantity detected among children with watery diarrhea in the Antibiotics for Children with severe Diarrhea (ABCD) trial.

	Ratio of risk ratios (95% confidence interval)	
Pathogen	Diarrhea on day 3	Rehospitalization or death by day 90
*Shigella*	0.87 (0.77, 0.97)	0.93 (0.76, 1.13)
*V. cholerae*	0.89 (0.68, 1.18)	0.96 (0.45, 2.05)
tEPEC	0.93 (0.85, 1.02)	0.98 (0.84, 1.13)
*Cryptosporidium*	0.94 (0.85, 1.03)	0.87 (0.73, 1.04)
ST-ETEC	0.94 (0.86, 1.02)	1.00 (0.86, 1.15)
*Giardia*	0.99 (0.87, 1.13)	1.14 (0.95, 1.38)
*E. bienuesi*	1.00 (0.74, 1.36)	0.70 (0.43, 1.15)
Sapovirus	1.00 (0.88, 1.12)	0.93 (0.74, 1.16)
EAEC	1.01 (0.93, 1.09)	0.87 (0.76, 1.01)
Astrovirus	1.06 (0.92, 1.21)	0.99 (0.76, 1.29)
Norovirus GII	1.06 (0.92, 1.21)	1.04 (0.83, 1.30)
*Campylobacter jejuni*/*coli*	1.08 (0.97, 1.19)	1.01 (0.85, 1.21)
Adenovirus 40/41	1.09 (0.98, 1.23)	1.13 (0.96, 1.33)
LT-ETEC	1.10 (0.91, 1.35)	1.17 (0.92, 1.50)
Rotavirus	1.16 (1.06, 1.26)	0.93 (0.78, 1.12)

In contrast, azithromycin treatment response did not vary with the quantity of *Campylobacter jejuni*/*coli*, LT-ETEC, and EAEC (Figure 1 and Table 1). For *C. jejuni*/*coli* diarrhea risk increased with pathogen quantity in both the control and placebo groups on day 3 (Figure 1A). This resulted in risk ratios for diarrhea on day 3 closer to the null at high *C. jejuni*/*coli* quantities. For LT-ETEC, diarrhoea risk on day 3 was very similar between azithromycin and placebo groups at high quantities, while for EAEC, the risk ratio for diarrhea on day 3 was independent of pathogen quantity.

**Figure 2:**
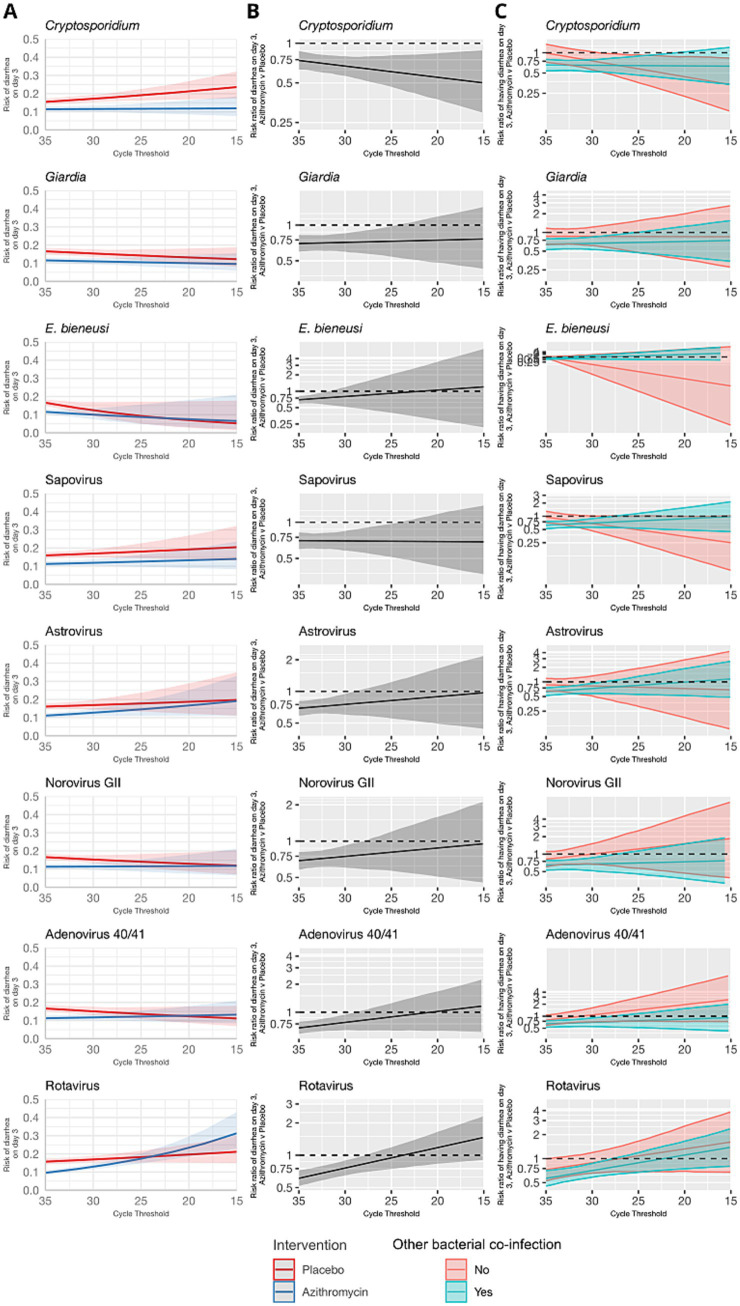
Modification of the effect of azithromycin on the risk of diarrhea on day 3 by non-bacterial pathogen quantities detected. For each non-bacterial pathogen, **(A)** the model-predicted risk of diarrhea on day 3 by pathogen quantity and treatment arm (red = placebo; blue = azithromycin), conditional on sample collection at day 0 and no co-detections. **(B)** The risk ratio for azithromycin compared to placebo for diarrhea on day 3 by pathogen quantity adjusted for the day of diarrhea on which the sample was collected and co-detection of other pathogens. **(C)** The adjusted risk ratio for azithromycin compared to placebo for diarrhea on day 3, stratified by the presence of a bacterial co-etiology (green = yes; red = no). Pathogen quantity is specified by the cycle threshold value from qPCR (i.e., smaller cycle thresholds correspond to higher pathogen quantity detected). Bands on all plots indicate 95% confidence bands.

The azithromycin treatment response was either inversely associated or not associated with pathogen quantity detected for viruses and parasites (Figure 2). Higher quantities of rotavirus, norovirus GII, adenovirus 40/41, and astrovirus, were associated with risk ratios for diarrhea on day 3 closer to the null. No consistent pattern was observed when stratifying by presence of a bacterial co-etiology. *Cryptosporidium* was an exception; higher quantities were associated with a stronger azithromycin treatment response. This was not explained by the presence of a bacterial co-etiology. Rather, the azithromycin treatment response was more strongly associated with *Cryptosporidium* quantity for episodes without a bacterial co-etiology.

**Figure 3:**
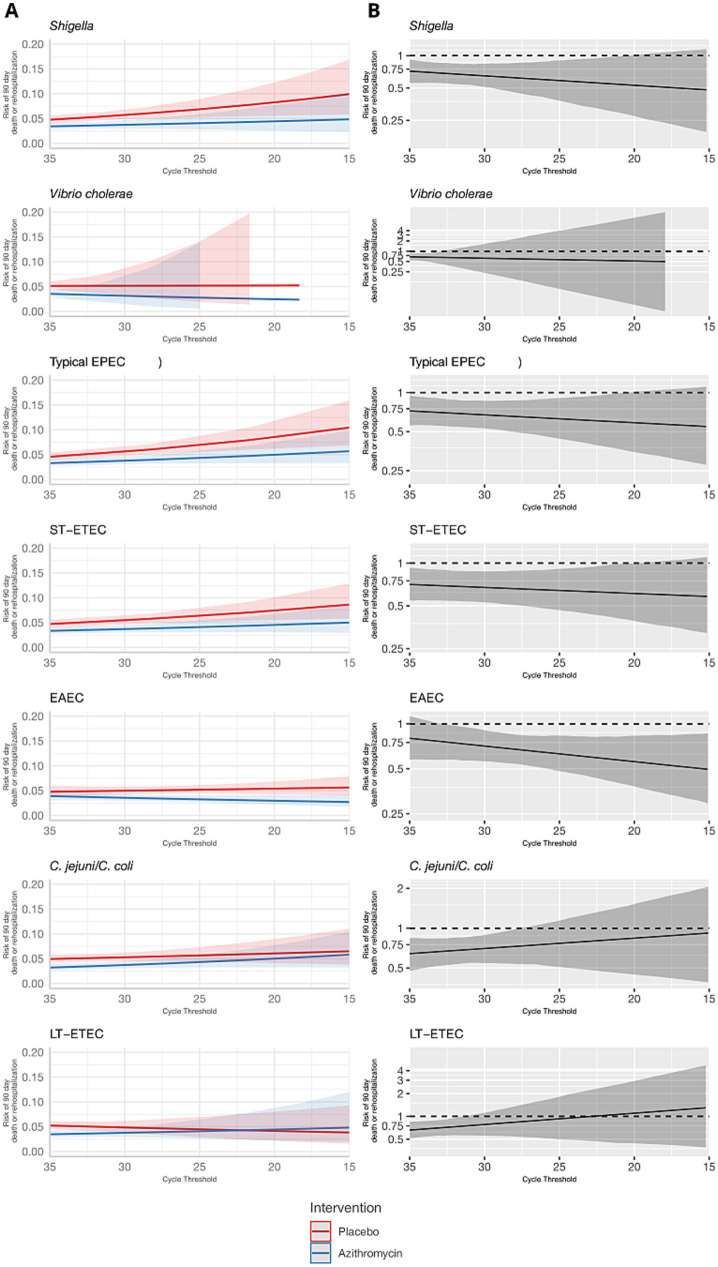
Modification of the effect of azithromycin on rehospitalization or death by day 90 by bacterial pathogen quantities detected. For each bacterial pathogen, **(A)** the model-predicted risk of rehospitalization or death by pathogen quantity and treatment arm (red = placebo; blue = azithromycin), conditional on sample collection at day 0 and no co-detections. **(B)** The risk ratio for azithromycin compared to placebo for rehospitalization or death by pathogen quantity adjusted for the day of diarrhea on which the sample was collected and co-detection of other pathogens. Pathogen quantity is specified by the cycle threshold value from qPCR (i.e., smaller cycle thresholds correspond to higher pathogen quantity detected). Bands on all plots indicate 95% confidence bands.

**Figure 4:**
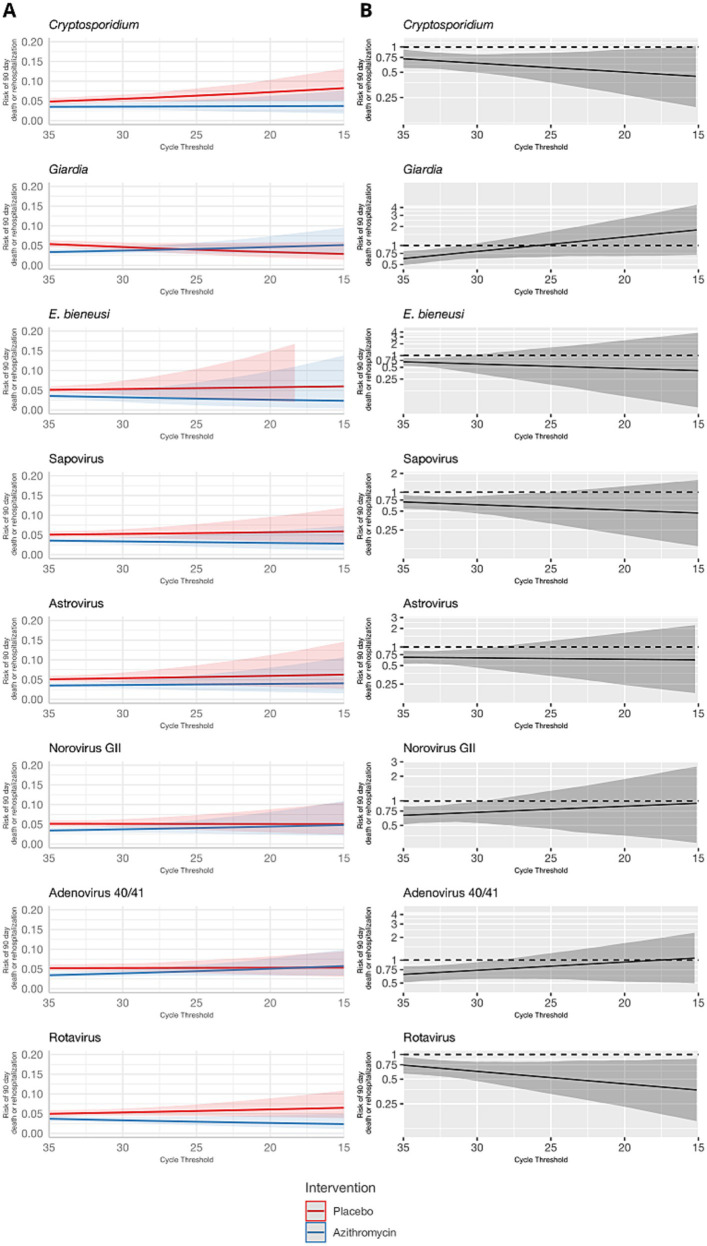
Modification of the effect of azithromycin on rehospitalization or death by day 90 by non-bacterial pathogen quantities detected. For each non-bacterial pathogen, **(A)** the model-predicted risk of rehospitalization or death by pathogen quantity and treatment arm (red = placebo; blue = azithromycin), conditional on sample collection at day 0 and no co-detections. **(B)** The risk ratio for azithromycin compared to placebo for rehospitalization or death by pathogen quantity adjusted for the day of diarrhea on which the sample was collected and co-detection of other pathogens. Pathogen quantity is specified by the cycle threshold value from qPCR (i.e., smaller cycle thresholds correspond to higher pathogen quantity detected). Bands on all plots indicate 95% confidence bands.

These results were consistent for the combined outcome of rehospitalization or death by day 90, but the associations were less precise and none of the effect heterogeneity was statistically significant (Table 1 and Figures 3, 4). The risk of death or hospitalization was 4.5% overall, 3.6% in the azithromycin group and 5.3% in the placebo group (risk ratio: 0.68, 95% CI: 0.54, 0.86). For episodes in which *Shigella* was detected at a higher quantity (Ct = 25), azithromycin was associated with a 57% reduction (95% CI: 29, 70%) in rehospitalization or death, compared to a 44% reduction (95% CI: 37, 54%) for episodes with *Shigella* detected at a lower Ct = 30 quantity. The only discrepant result was for rotavirus, for which higher quantities were associated with slightly stronger azithromycin treatment response.

Using the azithromycin treatment response as a probe to attribute etiology, we estimated that 4.1% of episodes could be attributed to *Shigella*, 3.0% could be attributed to ST-ETEC, 2.9% could be attributed to tEPEC, and 0.4% could be attributed to *Vibrio cholerae*. The population attributable fractions for *Campylobacter jejuni*/*coli*, EAEC, and LT-ETEC were zero.

**Figure 5:**
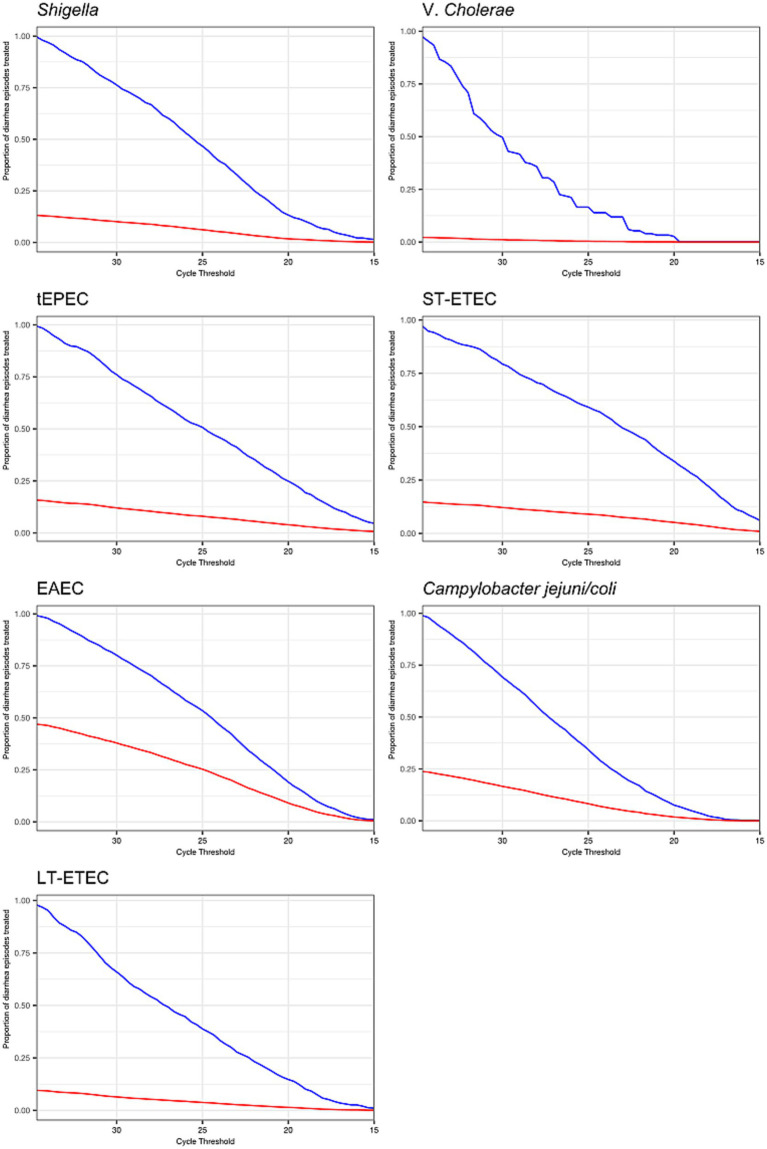
Prevalence of antibiotic treatment under decision rules that use cycle threshold cut-offs to define etiology and assign treatment. The proportion of diarrhea episodes overall (red line) and diarrhea episodes with the pathogen detected at any quantity (blue line) that would be treated with antibiotics if a given cycle threshold were chosen as the cut-off to define etiology and assign treatment.

The proportion of etiology-specific and overall diarrhea episodes that would be treated if treatment decisions were based on pathogen quantity cut-offs depended on the distribution of pathogen quantities among etiology-specific episodes and the distribution of etiologies (Figure 5). For example, treating all episodes with tEPEC at Ct ≤25 would result in 50% of episodes with tEPEC treated and 8% of episodes treated overall. Equally, 50% of episodes with tEPEC detected would be missed and left untreated. In contrast, treating all episodes with *V. cholerae* detected at a Ct ≤25 would result in only 16.6% of episodes with *V. cholerae* treated (82.4% untreated) and 0.4% of episodes treated overall.

## Discussion

In this reanalysis of qPCR data from the ABCD study, the azithromycin treatment response on diarrhea at day 3 was stronger among episodes with higher detected pathogen quantities for some of the most common bacterial causes of diarrhea: *Shigella*, *V. cholerae*, tEPEC, and ST-ETEC. This supports that these bacteria are likely the true etiology of diarrhea when detected at high pathogen quantities. While not statistically significant for *V. cholerae*, tEPEC and ST-ETEC, the observed heterogeneity in effects by pathogen quantity was largely consistent with previous comparisons of qPCR data between diarrhea cases and non-diarrheal controls from GEMS, which showed strong quantity dependent associations between all these bacterial pathogens and diarrhea, with the exception of tEPEC; the highest quantities of tEPEC were only moderately associated with diarrhea in GEMS (Liu et al., 2016). However, tEPEC was strongly associated with mortality in GEMS, providing additional support for tEPEC as an important cause of diarrhea (Levine et al., 2020).

Our data suggest that the relationship between treatment response and bacterial pathogen quantity is approximately linear, making the assignment of a definitive cycle threshold cut-off for diarrheal etiology difficult. Such a cut-off would be needed to define antibiotic treatment decision rules (i.e., treat if quantity < threshold and not if quantity > threshold) as well as to define case definitions for studies with pathogen-specific outcomes (e.g., *Shigella-*attributed diarrhea for a *Shigella* vaccine trial). Pathogen quantity could however be used to guide treatment decisions, for example by choosing to treat at a quantity threshold corresponding to a clinically meaningful treatment benefit if a derivative of this assay becomes available as a point of care test. The choice of threshold should also consider the potential benefits of expanded use of azithromycin to treat episodes of bacterial diarrhea in the context of the growing azithromycin resistance rates in key Gram-negative bacteria, including *Shigella* spp. (Baker et al., 2015; Nuzhat et al., 2022).

The lack of associations between pathogen quantity and azithromycin treatment response for *Campylobacter*, LT-ETEC, and EAEC were consistent with the weaker associations between pathogen quantities and diarrhea observed for these pathogens in MAL-ED and GEMS (Liu et al., 2016; Platts-Mills et al., 2018). These prior studies of diarrhea etiology attributed relatively few diarrhea episodes to these pathogens despite the fact that they were commonly detected during diarrhea, and our results corroborate that they may be relatively rare causes of diarrhea in children in low-resource settings who have early and high levels of exposure. Sub-clinical carriage of *Campylobacter* is frequent, complicating the assignment of a cut-off to attribute diarrhea to this pathogen (Houpt et al., 2021) low qPCR cut-offs used to attribute *Campylobacter* diarrhea based on MAL-ED and GEMS, for example used in the previous analysis of the ABCD study, have been criticized as potentially being poorly sensitive (Pavlinac et al., 2024). However, the lack of treatment response even among episodes with high *Campylobacter* quantity support the use of a low Ct (i.e., high quantity) cut-off for attributing *Campylobacter* etiology.

A key purpose of vaccine probe studies is to identify the proportion of disease attributable to the vaccine-targeted pathogen (Feikin et al., 2014). In this “antibiotic probe” study analogue, we estimated population attributable fractions for each of the bacterial pathogens based on the azithromycin treatment response at observed pathogen quantities for each diarrhea episode. The resulting relative ranking of pathogens matched that previously reported in GEMS and MAL-ED (Liu et al., 2016; Platts-Mills et al., 2018). However, the proportions attributable were smaller in magnitude, likely because azithromycin is not perfectly effective in preventing diarrhea on day 3. Importantly, no fraction of diarrhea could be attributed to *Campylobacter*, EAEC, or LT-ETEC using the azithromycin treatment response as a probe given the inverse associations between quantities of these pathogens and the treatment response. This is likely reflective of the fact these pathogens are relatively rare causes of diarrhea in our research setting.

We also predominantly observed an inverse association between non-bacterial (virus and parasite) quantities and treatment response at day 3. High viral quantities were strongly associated with poor treatment outcomes, suggesting the true etiology of these diarrheal cases was viral, as we would not expect an azithromycin treatment response in cases of viral diarrhea. The observation of improved treatment outcomes at lower virus quantities, may be due to the increased likelihood of bacterial co-infection at lower virus quantities, such that the virus was carried at sub-clinical levels while the etiology was truly bacterial.

Interestingly, higher quantities of rotavirus were associated with a small reduction in rates of death or hospitalization at day 90. Dehydration and/or undernourishment were inclusion criteria for the ABCD study. Malnourishment can suppress the immune system; the WHO recommends routine treatment of children with severe acute malnutrition (SAM) with a broad-spectrum antibiotic, and in some settings azithromycin administration, for SAM has been reported to improve recovery and reduce mortality rates (O’Brien et al., 2021). Studies conducted in both Africa and Asia have reported that rotavirus infection is associated with undernutrition (Das et al., 2017; Gasparinho et al., 2017). It is possible that the improved azithromycin treatment outcome observed for higher quantities of rotavirus in this study is due to the anti-inflammatory effects of azithromycin to treat malnutrition, which may have led to improved recovery from rotavirus infection (Zimmermann et al., 2018). Further, improved outcomes could also be a result of azithromycin-driven gut microbiota ablation, which has previously been shown to enhance humoral immunity and reduce severity of rotavirus diarrhea (Uchiyama et al., 2014).

For parasites, *Giardia* and *E. bieneusi* quantities were not determinants of the azithromycin treatment response, which was expected since parasites are not susceptible to azithromycin. Interestingly, *Cryptosporidium* was unique amongst the non-bacterial pathogens such that high quantities were associated with improved treatment response at both day 3 and day 90. This improvement was not explained by presence of bacterial co-infections but has been observed previously in a case series of HIV-infected adults (Kadappu et al., 2002). The mechanism for improved treatment outcome is unclear; however, it is possible that the immunomodulatory effects of azithromycin may again be contributing to improved recovery in *Cryptosporidium* infection.

This study has some limitations. We had no azithromycin susceptibility data from the bacterial pathogens tested for in this analysis; phenotypic resistance in commensal *E. coli* from a subset of ABCD participants from all sites was however 24% 3 months after enrolment (Ahmed et al., 2021). It is possible that azithromycin resistance amongst the Gram-negative bacterial pathogens reduced the effect of the azithromycin treatment. Notably, *Campylobacter* species have been reported to exhibit high levels of macrolide resistance globally. A recent systematic review of *Campylobacter* associated diarrhoea in humans reported macrolide resistance levels of 54% in Eastern Africa and 70% in Southern Africa (Hlashwayo et al., 2021). Although recent data on macrolide resistance in human diarrhoeal isolates from Asia are lacking, studies from poultry farms in Bangladesh report resistance levels >90% (Hasan et al., 2025). It is therefore plausible that azithromycin resistance amongst *Campylobacter* pathogens analyzed in this study may have contributed to the lack of association observed between *Campylobacter* abundance and treatment response.

Furthermore, we did not differentiate bacterial pathogens at the species level. Given the variation in virulence and antibiotic resistance profiles among species within the same genus, such as *Shigella* and *Campylobacter*, this could have introduced bias into our results.

Mechanistic studies of azithromycin have shown it exhibits immunomodulatory activity through the regulation of multiple inflammatory pathways (Zimmermann et al., 2018). Our results may have therefore been confounded by children who did not have bacterial attributable diarrhea but exhibited a treatment response due to the anti-inflammatory activity of azithromycin. In addition, this study of effect heterogeneity was underpowered since the primary ABCD trial was powered for population-level effects. While few estimates of heterogeneity were statistically significant, changes in the magnitude of the azithromycin effect remain informative. Finally, our dataset is derived from children enrolled in the ABCD study, which had restrictive enrolment criteria requiring all children to have dehydration or malnutrition. Approximately half of children presenting to ABCD recruitment sites and screened for ABCD recruitment were ineligible due to not being dehydrated or wasted (Ahmed et al., 2021). Therefore, the estimates of the proportions of children who would be treated under varying quantity cut-offs may not be generalizable to all children with diarrhea. Furthermore, the results may not be generalizable to high-income countries (HICs). *Campylobacter* spp. are endemic and show high sub-clinical carriage in LMICs but not in HICs (Epps et al., 2013). It is feasible that there may be a clearer association between *Campylobacter* quantity and azithromycin treatment response in HIC settings where *Campylobacter* is not endemic.

In this reanalysis of qPCR diagnostic data from the ABCD trial, the relationship between bacterial quantity and treatment response for *Shigella*, *V. cholerae*, tEPEC, and ST-ETEC support that attribution of diarrhea etiology to these pathogens is more accurate as pathogen quantity increases and suggest that the use of a pathogen quantity cut-off to attribute etiology is appropriate for observational and intervention studies of diarrhea using molecular diagnostics. However, because the heterogeneity of treatment response was linear with pathogen quantity, the appropriate qPCR cut-off for assigning etiology may vary by specific context.

## Data Availability

The data analyzed in this study is subject to the following licenses/restrictions: anonymized participant data will be made available on requests directed to the corresponding author. Proposals will be reviewed and approved by the sponsor, investigator, and collaborators based on scientific merit. After approval of a proposal, data can be shared through a secure online platform after signing a data access agreement. Requests to access these datasets should be directed to AC (deay@who.int).
