# Supplementary material for: Azithromycin treatment response as a probe to attribute bacterial etiologies of diarrhea using molecular diagnostics: a reanalysis of the Antibiotics for Children with Severe Diarrhea (ABCD) trial
Source: Front Microbiol. 2025 May 20;16:1606207. doi: 10.3389/fmicb.2025.1606207 (PMC12130904; doi:10.3389/fmicb.2025.1606207)
Supplement: Supplementary file 1 [file Data_Sheet_1.pdf]

Supplementary Materials

Azithromycin Treatment Response as a Probe to Attribute Bacterial Aetiologies of Diarrhoea using Molecular Diagnostics: A Reanalysis of the AntiBiotics for Children with severe Diarrhoea (ABCD) Trial.

Table S1. P-values for the interaction term between treatment arm assignment and the quantity of pathogen detected when specified with a linear and quadratic term among children with watery diarrhea in the AntiBiotics for Children with severe Diarrhoea (ABCD) Trial.

Pathogen	P-value of linear term from linear model	P-value of quadratic term from quadratic model
Vibrio cholerae	0.4	0.9
Rotavirus	0.001	0.03
Shigella/EIEC	0.02	0.2
ST-ETEC	0.1	0.08
Astrovirus	0.4	0.7
Cryptosporidium	0.2	0.3
Norovirus GII	0.4	0.7
Adenovirus 40/41	0.1	0.3
C. jejuni/C. coli	0.2	0.06
tEPEC	0.1	0.7
Sapovirus	0.9	0.02
E. bieneusi	0.5	0.6
EAEC	0.8	0.2
Giardia	0.8	0.2
LT-ETEC	0.1	0.3

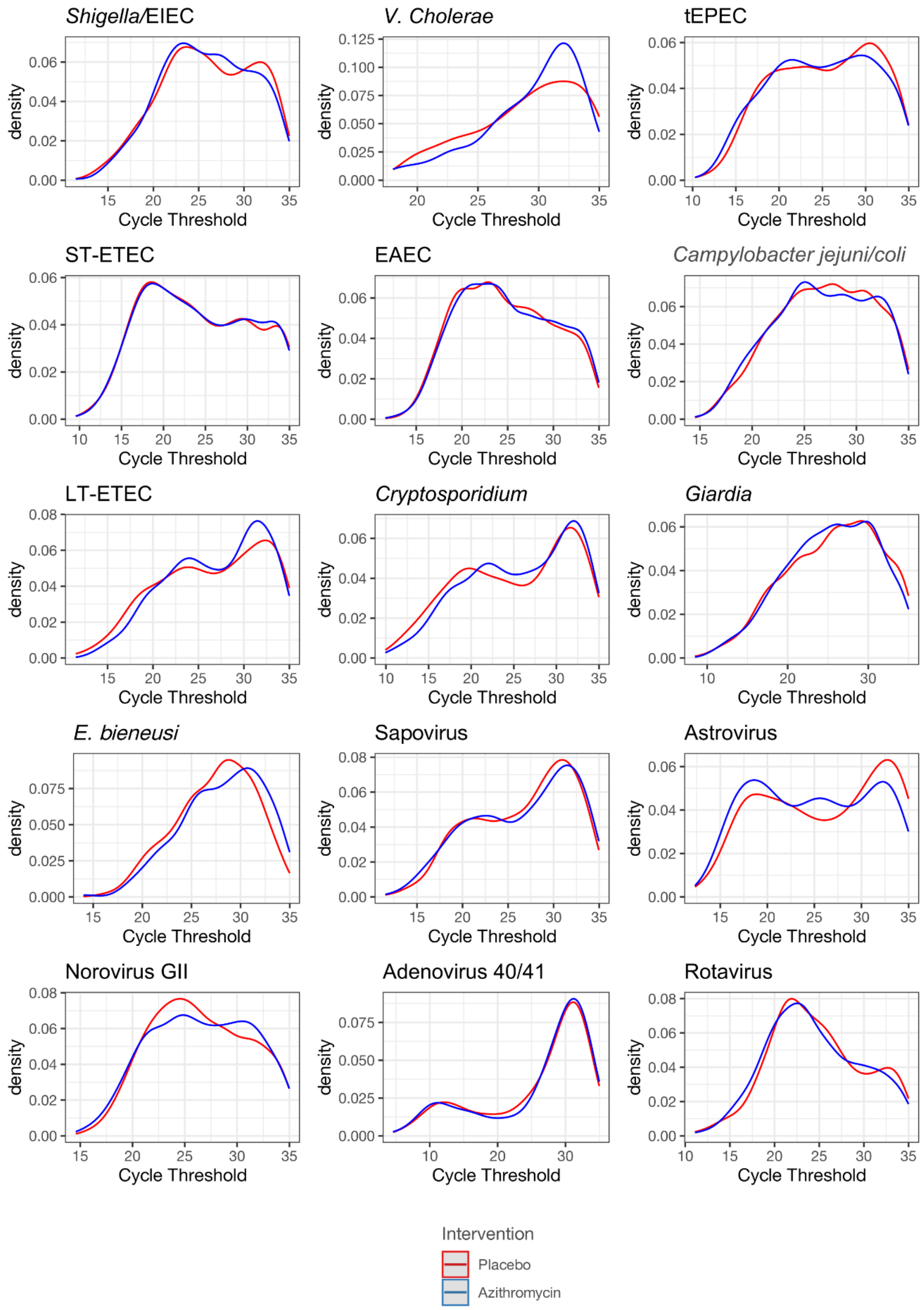

Figure S1. Density of pathogen quantities detected based on qPCR cycle threshold by intervention group (red = placebo; blue = azithromycin) in the ABCD trial.
